# Knowledge and Attitude of Iranian Medical University Students about Organ Donation and Transplantation: A Cross-Sectional Study

**DOI:** 10.4314/ejhs.v32i1.14

**Published:** 2022-01

**Authors:** Shohreh Kolagari, Javad Bayei, Vahid Asoodeh, Siamak Rajaee, Zahra Mehbakhsh, Mahnaz Modanloo

**Affiliations:** 1 Nursing Research Center, Golestan University of Medical Sciences, Gorgan, Iran; 2 Faculty of Nursing and Midwifery, Golestan University of Medical Sciences, Gorgan, Iran; 3 Department of Surgery, Faculty of Medicine, Golestan University of Medical Sciences, Gorgan, Iran; 4 Department of Biostatistics, Faculty of Health, Golestan University of Medical Sciences, Gorgan, Iran

**Keywords:** Knowledge, Attitude, Organ Transplantation, Students, Medical, Brain Death

## Abstract

**Background:**

Medical professionals' knowledge of and attitudes toward organ donation and transplantation have positive impact on donation rates. The aim of this study was to determine the knowledge and attitude of medical university students in Iran about organ donation and transplantation.

**Methods:**

This cross-sectional study was carried out on 1078 undergraduate students in Golestan University of Medical Sciences, Gorgan, Iran, from January to June 2019. All eligible students were recruited using convenient sampling. Data were gathered using knowledge and attitude toward organ donation and transplantation questionnaire. The higher scores of both subscales, the knowledge subscale (range from 0 to 13) and the attitude subscale (range from 13 to 65), indicate the better knowledge and attitude toward organ donation and transplantation.

**Results:**

The mean age of students was 22.24±5.16 years. Finding showed that the mean score of students' knowledge and attitude toward organ donation and organ transplantation were 8.48±1.71 and 48.55±8.11 respectively. Also, the mean score of students' knowledges in females (P<0.001), married (P=0.001) and who had organ donation card (P<0.001) was significantly higher. Nearly all of the students had heard about organ donation (98.3%) and organ transplantation (98.4%). Majority of them pointed that their source of information about organ donation and transplantation was television (TV) program (47.1%). Most of the students (73.8%) reported that they agree to donate their organs but most of them (67.5%) did not know how to obtain organ donation card and only 9.6% of them had the organ donor card as a volunteer donor.

**Conclusion:**

According to finding, despite the awareness and favorable attitude about organ donation and transplantation among medical students, the number of registered donors was low. In addition to promoting college students' awareness about organ donation for increasing registered donors, it is needed to aware and pursue lay people through social media.

## Introduction

Organ transplantation is a significant development in management of end organ failure that can cause noticeable improvements in the quality of life (1) and life expectancy (2) in patients who are waiting for life-saving organ transplants. Although transplantable organs and tissues can be supplied by alive or deceased donors (3), the shortage of donors is a worldwide issue and many patients with organ failure die every year on transplant waiting lists because of this shortage (4). To overcome the high demand of organ transplantation, brain dead patients are the most reasonable resources of organ transplant (5).

In Iran the rate of organ transplantation is lower than Europe and the United States (US), due to shortage of donors. In 2017 in some European countries such as Spain, Portugal and Belgium, the average number of post-mortem donors was 46.9, 34 and 33.6 person per million donors, respectively, ranking first to third in the world. The US ranked fifth in the world with an average of 32 person per million donors; while Iran ranks thirty-fifth (6). In Iran, 5 to 8 thousand brain deaths occur each year; but just 10% are donated to the patients who are on the national transplant waiting list (7). According to the report of Iranian organ donation association, in Iran 26,000 people are waiting for an organ transplant. One person being added to the waiting list every 10 minutes, and every 2 hours, a patient dies in need of an organ. However, each brain-dead person can save eight lives from death and improve the quality of life of 53 patients (8).

Despite the importance and vitality of the issue, transplantation medicine is one of the most challenging and complex areas of modern medicine. Organ donation is a complex and multifactorial issue, involving factors such as personal beliefs, ethical, legal, medical, cultural, organizational and societal factors (9, 10). Individuals' knowledge and attitudes toward organ donation and transplantation can influence on their behavior or intention to behave (11). Knowledge and attitudes of healthcare providers regarding organ donation and transplantation are known as factors that can increase donation rates by encouraging brain dead patients' family to donate organs of their family members (10, 12, 13).

Making decision for organ donation is an important and complicated decision for own person and his family that the healthcare providers have a critical role in this issue and their knowledge and attitude may lead to facilitating the donation process through influencing decision-making process of the family members of brain-dead patients. (14). Medical university students, as future health care providers, are perceived to have a significant role in this regard. Therefore, determining the level of their knowledge and attitudes towards organ donation and transplantation are important (15). Therefore, this study aimed to determine the level of knowledge and attitude of a medical university students in northeast Iran, toward organ donation and transplantation.

## Methods

This cross-sectional study was conducted in Golestan University of Medical Sciences, Gorgan, northeast of Iran, from January to June 2019. All undergraduate students, who met the inclusion criteria were recruited using census sampling. The inclusion criteria were, no history of employment in the organ donation and transplantation center and no experience of participating in the workshop related to organ donation and transplantation. At the initial contact, the aim of the study and voluntary participation in the study was explained for each student and they were ensured about confidentiality of their information. A total of 1078 students who met the inclusion criteria, were agreed to participate in the study and signed the informed consent forms.

Data were collected using a questionnaire comprised demographic characteristics (included age, gender, marital status, field of study, year of study and source of information) and knowledge and attitude toward organ donation and transplantation. The questionnaire used for evaluation of students' knowledge and attitude toward organ donation and transplantation has two subscales including knowledge (questions 1–13) and attitude (items 14–26) subscales. Responses to the items dealing with knowledge subscale were based on a two-point scale, ‘Yes’ or ‘No’. The ‘Yes’ response was given a score of 1 and the ‘No’ response was given a score of 0. Reverse scoring was done for questions 6, 9 and 10. The scores of the knowledge subscale ranged from 0 to 13. The higher the scores, the more knowledge toward organ donation and transplantation. The attitude subscale was rated on a 5-point likert scale for determining levels of agreement (from strongly disagree=1, to strongly agree=5). Items 7 and 8 scored in the reverse order. The scores of attitude subscales range from 13 to 65. The higher the scores, the better attitude toward organ donation and transplantation (4). A study in Iran confirmed the reliability of this questionnaire, with Cronbach alpha of 76.3 (16). Additionally, due to the specific cultural and religious context in Iran, three open-ended questions were asked from participants regarding religious, cultural and legal issues about organ donation and transplantation.

**Sample size**: Estimation of sample size was based on previous study (17). At a level of α=.05 with a power of 0.8, we calculated that it was necessary to enroll 1000 eligible participants for this study, so in order to compensate for possible drop outs, we planned to recruit 1080 participants.

**Statistical analysis**: Data analysis was performed using the statistical package for social sciences (SPSS) software (IBM Inc, Chicago, Il, USA) version 16.0 using Mann-Whitney and independent t-test. The normality of the quantitative data distribution was assessed using Shapiro-Wilk test. P-value<0.05 was considered statistically significance.

## Results

The mean age of participants was 22.24±5.16 years. The findings showed that majority of students were female (60.7%) and single (85.3%). Most of them reported that they did not have any history of brain death (93.1%) or organ transplantation (93.9%) in their family or relatives. Only 9.6% of participants registered to be a donor and had the organ donor card. Majority of them pointed that their most source of information about organ donation and transplantation was television (TV) programs (47.1%) and 12.6% of them reported that they did not use any source of information at all (Table 1).

**Table 1:** Socio-demographic characteristics of the students (n=1078)

Demographic characteristics		N	%
Gender	Male	424	39.3
	Female	654	60.7
Marital status	Single	919	85.3
	Married	159	14.7
Residential status	Urban	991	91.9
	Rural	87	8.1
Field of study	Medicine	336	31.2
	Dentistry	120	11.1
	Nursing	238	22.1
	Midwifery	58	5.4
	laboratory science	68	6.3
	Anesthesia	41	3.8
	Nutrition science	50	4.6
	Operating room science	53	4.9
	Environmental health	50	4.6
	Public health	64	5.9
History of brain death in family or relatives	Yes	74	6.9
	No	1004	93.1
History of organ transplantation in family or relatives	Yes	65	6.0
	No	1012	93.9
Having the Organ Donor Card	Yes No	104 974	9.6 90.4
Source of information	TV	508	47.1
	Website	83	7.7
	Social network	246	22.8
	Magazine Friends & relative	1 83	0.1 7.7
	Book	20	1.9
	Nothing	136	12.6
Grade (Year)	1^st^	397	36.8
	2^nd^	388	36
	3^rd^	293	27.2

The findings showed that the mean score of students' knowledge toward organ donation and transplantation was 8.48±1.71. Nearly all of respondents had heard about organ donation (98.3%) and organ transplantation (98.4%) and 93.3% of them knew that brain dead patient's organs can be donated to organ recipients. But most of them (67.5%) did not know how to obtain organ donation card. Of the participants, 73.5% thought that Hepatitis B or C carriers are prevented to donate all of their solid organs. Furthermore, 66.8% thought that risk of getting cancer in organ recipients is higher than others (or organ reception is a risk factor of cancer) (Table 2).

**Table 2:** The frequency of students' knowledge toward organ donation

Question	Yes		No	

	N	%	N	%
1. Have you heard of the term “organ donation?”	1060	98.3	18	1.7
2. Have you heard of the term “organ transplantation?”	1061	98.4	17	1.6
3. Are you aware of “transplantation of human organs act?”	354	32.8	724	67.2
4. Do you know where you can obtain organ donation cards?	350	32.5	728	67.5
5. Can a brain-dead patient's organs be donated?	1006	93.3	72	6.7
6. Will certified brain-dead registered organ donor be immediately disconnected from ventilation support?	568	52.7	510	47.3
7. Can parents/guardians make substitute decision making for mentally disabled persons in the regard of organ donation?	703	65.2	375	34.8

8. Donor's and recipient's blood group MUST be matched?	989	91.7	89	8.3
9. Donor's human leukocytes antigen MUST be identical to that of the recipient for any organ transplantation?	759	70.4	319	29.6
10. Hepatitis B and C carriers can donate all of their solid organs except the liver organs?	286	26.5	792	73.5
11. Malignancy is always a contraindication to cadaveric organ donation?	548	50.8	530	49.2
12. Increased risk of opportunistic infections is a common complication to all transplantations?	734	68.1	344	31.9
13. Organ transplant recipients are more prone to developing of cancer after transplantation?	720	66.8	358	33.2

Also, findings showed that the mean score of students' attitudes toward organ donation and organ transplantation was 48.55±8.11. 73.8% of respondents reported their agreement about donating their organs after death. However, 42.3% indicated positive attitude toward donating their family members' organs. 34.2% were neutral and 23.5% disagreed to donate their family members' organs. They were asked about time of termination of medical treatment for registered organ donors, nearly 50% had no idea about this issue (Table 3).

**Table 3:** The frequency of students' attitude toward organ donation

Items	Strongly agree		Agree		No idea		Disagree		Strongly disagree	
	
	N	%	N	%	N	%	N	%	N	%
14. I encourage the family of brain death patients to donate his/her organs	350	32.6	389	36.2	300	27.9	22	2.0	13	1.2
15. I feel comfortable to think or talk about organ donation	227	21.1	323	30.0	372	34.6	120	11.2	33	3.1
16. I agree to donate organs when I die	482	44.8	312	29.0	181	16.8	58	5.4	42	3.9
17. I agree to donate my family member's organs	217	20.2	237	22.1	367	34.2	148	13.8	104	9.7
18. I think donating one's organ adds meaning to one's life	443	41.4	358	33.5	226	21.1	28	2.6	14	1.3
19. My religion agrees with organ donation or transplantation	430	40.1	319	29.8	265	24.7	29	2.7	28	2.6
20. I fear that my body will be disfigured, if I donate organs	73	6.8	156	14.6	394	36.8	251	23.4	198	18.5
21. I think there will be premature termination of medical treatment for registered organ donors	81	7.6	185	17.5	524	49.5	162	15.3	107	10.1
22. I think alive organ donation is better than cadaveric organ donation in solving shortage	113	10.6	218	20.4	460	43.1	165	15.5	111	10.4
23. I believe lasting life of one's organ in another's body	329	30.7	472	44.0	209	19.5	44	4.1	18.0	1.7
24. The possibility of lasting the life of an organ needful person makes me feel comfortable	329	30.7	415	38.7	286	26.7	26	2.4	15	1.4
25. Lasting the life of an organ needful person with donated organ is a God-willing work	526	49.3	389	36.5	136	12.7	7	0.7	9	0.8
26. Donating organ to a needful person makes donor's soul pleased	481	45.1	339	31.8	213	20.0	11	1	22	2.1

Data analysis revealed that the mean score of knowledge toward organ donation in females was significantly higher than males (P<0.001). Single participants had a better knowledge in comparison with married participants (P=0.001). The participants who registered as an organ donor and had organ donation card, had significantly better knowledge and attitude in regard with organ donation (P<0.001). Comparison of the students' attitude showed that females had more positive attitudes than males (P<0.001). The attitude of the students who lived in rural area, were less positive than the students who were lived in the urban areas (P=0.002). Finding indicated that students in second grade had the most positive attitude toward organ donation (P=0.028). However other variables (field of study, history of brain death in family or relatives and history of transplantation in family or relatives) did not show significant relationship with knowledge and attitude of medical university students.

The analysis of three open-ended questions showed that the majority of respondents had positive attitude toward organ donation. Most of them (94.4%), expressed that their religion was not an opponent of transplanting any organs from any gender to another. Of the participants, 65.1% agreed to donate organs of an unidentified person or a person who has no family and relatives to save one's life. Most of them pointed that saving one's life is the most precious issue. The results showed that 82.6% of participants believed that the organs of a person who has organ donation card, should be donated without one's family consent and they believed that final decision maker should be the own person before dying. However, 17.4% believed that family consent should be considered (Table 4).

**Table 4:** The students' point of view toward organs donation regarding cultural and legal issues

Question		N	%
1. Is transplanting any organ from a man to a woman and vice versa in contrast with your religion?	Yes	58	5.6
	No	978	94.4
2. Is it true if we donate organs of an unidentified person or a person without family to save one's life?	Yes	669	65.1
	No	359	34.9
3. Now in Iran if someone who has organ donation card, die, his or her organs couldn't be donated without permission of his or her family. In your opinion person's decision should be performed or family decisions?	Person	863	82.6
	Family	182	17.4

## Discussion

Making decision for organ donation is an important and complicated issue for each person and his family. Knowledge and attitude of each person toward organ donation can play a key role in this complicated process (17). Therefore, it is important that health care providers have enough knowledge and attitude to inform people and facilitate this process (4, 5, 18). In line with the results of present study, the results of a study conducted among undergraduate health science students in Ethiopia revealed that undergraduate health science students have a good knowledge and a positive attitude toward organ donation (18). However, the results of a study in Saudi Arabia indicated a low level of knowledge about organ donation among medical and health sciences students (19). In addition, another study by Darlington et al. indicate a poor knowledge regarding organ donation among Indian medical students (5). These differences may be because of different questionnaires or different culture of participants.

According to the findings, most of the students agreed to donate their organs after death. However, 42.3% showed desire to donate their family member organs. It indicates that there are some gaps between attitude and behavior of students especially when someone facing an emotional challenge. The results of a study conducted by Montero Salinas et al. among hospital staffs are consistent with the results of present study (20). The results of another study by Taghizadeh et al. on 5^th^ and 6^th^ year medical students of Tabriz University of medical sciences revealed that most of the students were willing to donate their family member organs. However, their tendency to donate the organs of their family members was lower than the tendency of donating their own organs (21). Furthermore, an interventional study was conducted with aim to investigate the effects of phased education on attitudes of people concerning organ donation and willingness to donate organs after brain death. Before education, the tendency of donating family member's organs was significantly lower than donating one's own organs. After education, their intention for both family members and their organs significantly increased. However, the intention of donating family member organs remained significantly lower than donating one's own organs (22). This can state that changing attitudes toward organ donation is a time-consuming process and it needs to be internalized overtime.

The findings of current study showed that organ donor card holders had significantly better knowledge and attitude toward organ donation in comparison with who didn't. This result was consistent with the findings of Mohebi et al. study on medical sciences students (17). Also, a study was carried out on health care professionals by Emami et al, showed a significant correlation between having organ donor card, history of blood donation, history of donation around participants and the performance of healthcare professionals (23).

The results of current study indicated that there is a significant relationship between gender and level of knowledge and attitude. In a way that females showed higher knowledge and more positive attitudes toward organ donation. This result is consistent with the findings of Almutairi et al. study in Saudi Arabia (19). In the contrary, the results of the study by Wolide et al. indicated that male students had higher knowledge score (18). However, other studies did not confirm any significant relationship between gender and knowledge of participants about organ donation (17, 24–26).

The present study illustrated that the most used source of information regarding organ donation and transplantation was TV. The results of several studies done in Iran and other foreign studies were consistent to this study and in all of them TV was found as the main source of information (17, 19, 27–34). The results of the study carried out by Manzari et al. among families with brain dead patient, pointed to the effective role of TV in decision-making of family regard donating their family member organs (35). In addition, most of the participants in the study of Amani et al. stated that mass media play a key role in encouraging people to donate and institutionalize the culture of organ donation (36). This shows the determinative role of TV in increasing knowledge and attitudes of population toward organ donation and transplantation.

Organ donation and transplantation is fast becoming a serious bioethical dilemma from the view point of healthcare providers despite of having regulations for over sighting the organ donation and transplantation. Making decision for unidentified persons or persons who have no family and relatives is an ethical dilemma regarding organ donation. In Iran having organ donor card is not valid for donating solid organs from the dead body and official written testament or family members written informed consent is necessary (37). So organ donation acts suggested to be revised at least for unidentified persons.

This study has some limitations. First, the crosssectional design of the present study limits our ability to form firm conclusions regarding causality. Second, the result of this study may not be generalizable to all medical university students in the country or internationally.

In conclusion, it seems that despite the awareness and favorable attitude about organ donation and transplantation among medical university students, the number of medical students registered donors was low. It is suggested to implement effective methods such as workshop to change students' beliefs toward organ donation and internalization of positive beliefs. In addition, for pursuing the desire of people for organ donation, it is needed to aware them through social media especially TV considering their cultural, religious and social characteristics.
